# Right Coronary Artery to Coronary Sinus Fistula Post Endomyocardial Biopsy: A Case Report of a Rare Complication

**DOI:** 10.1155/2020/7914737

**Published:** 2020-03-01

**Authors:** Aniket S. Rali, Farhad Sami, Andrew Sauer, Zubair Shah

**Affiliations:** ^1^Department of Cardiovascular Medicine, The University of Kansas Health System, USA; ^2^Department of Internal Medicine, The University of Kansas Health System, USA

## Abstract

Endomyocardial biopsy (EMB) continues to remain the gold standard for surveillance of rejection post orthotropic heart transplantation (OHT). It can be performed under fluoroscopic or echocardiographic guidance. In the hands of an experienced operator, the complications of EMB are uncommon with <1% chance of any serious acute complications. Most common complications of EMB include access site-related complications, namely, venous thrombosis, carotid cannulation, hematoma, air embolism, and pneumothorax. We present a case of a rare complication of EMB in a patient with OHT causing a coronary sinus to right coronary artery (RCA) fistula.

## 1. Introduction

Endomyocardial biopsy (EMB) continues to remain the gold standard for surveillance of rejection post orthotropic heart transplantation (OHT). ACC/AHA and ESC recommend EMB as a diagnostic tool in the evaluation and treatment of unexplained cardiomyopathy encompassing 14 different clinical scenarios [1, 2]. Since the first intravascular approach of EMB described in 1962 by Sakakibara and Konno [3], significant evolution in techniques and approaches has taken place [4]. Currently, EMB is done via an internal jugular or femoral vein (or the femoral artery if getting left heart biopsy) utilizing transcatheter insertion of a bioptome to biopsy the endocardium of the interventricular septum. It can be performed under fluoroscopic or echocardiographic guidance. In the hands of an experienced operator, the complications of EMB are uncommon with <1% chance of any serious acute complications [5, 6]. Most common complications of EMB include access site-related complications, namely, venous thrombosis, carotid cannulation, hematoma, air embolism, and pneumothorax [7, 8]. Cardiac specific complications have also been described and include self-limited arrhythmias, myocardial perforation, pericardial tamponade, pulmonary embolization, and tricuspid valve damage [5]. We present a case of a rare complication of EMB in a patient with OHT causing a coronary sinus to the right coronary artery (RCA) fistula.

## 2. Timeline

A 63-year-old male patient underwent heart transplantation on for Stage D heart failure due to ischemic cardiomyopathy. The patient continued to have unremarkable hospital course posttransplant and was discharged to home. Over the next 12 months, the patient continued to have an uncomplicated course with normal EMBs free of rejection. At our institute, we perform weekly biopsies for the first month posttransplant and then start spacing them out monthly for the first year. Per our institutional protocol, the patient presented for a coronary angiography, right heart catheterization, and EMB one year after transplant. We perform all our EMBs for transplanted hearts under fluoroscopy guidance. During this visit, he was noted to have a large RCA with the arteriovenous fistula to coronary sinus on coronary angiography. Follow-up coronary CTA confirmed a RCA to coronary sinus fistula. A retrospective review of all the EMB specimens obtained till date was performed and showed one of the biopsy samples containing blood vessel tissue.

## 3. Case Presentation

**Figure 1:**
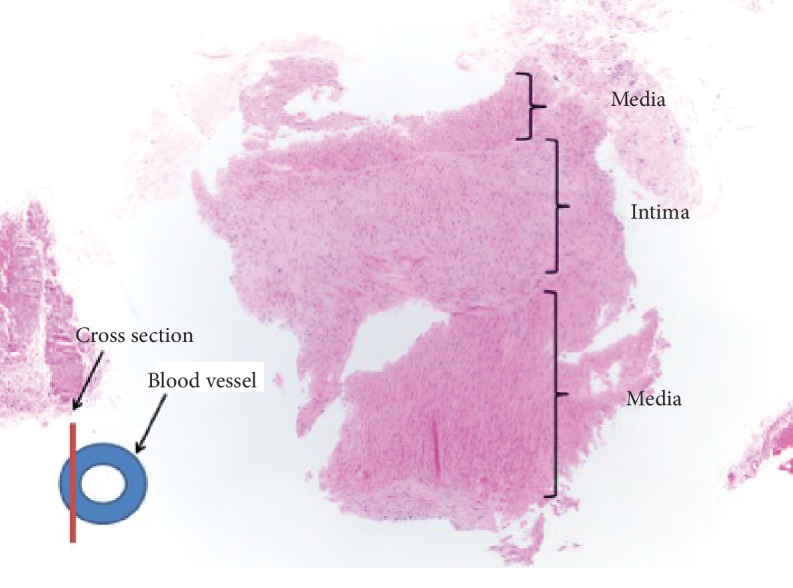
EMB pathology specimen confirming a blood vessel.

Our patient is a 63-year-old male who underwent heart transplantation for stage D heart failure with reduced ejection fraction (HFrEF) due to ischemic cardiomyopathy. The patient's postoperative course was unremarkable, and there was no evidence of allograft rejection on the 12 biopsies obtained within first six months posttransplantation. He presented for an outpatient right heart catheterization (RHC), coronary angiography, and EMB for his routine one year posttransplantation evaluation. Coronary angiography revealed a very large RCA measuring 5 mm with a fistulous connection from posterolateral branch to possibly the right atrium (Supplemental [Supplementary-material supplementary-material-1]). There was no “step-up” in venous saturations suggestive of an intracardiac shunt. Hemodynamics were unremarkable with a pulmonary artery pressure of 29/18, a pulmonary capillary wedge pressure of 12 mmHg, and a cardiac output of 5.6 L/min. The patient's transthoracic echocardiogram (TTE), two months posttransplant, was suggestive of a very subtle right coronary artery to right atrial shunt which was not noted on the initial read (Supplemental [Supplementary-material supplementary-material-1]). This anomaly was not present on the donor heart TTE immediately pretransplant. A retrospective systematic review of all EMB slides revealed a blood vessel sample present on one of the prior biopsies (Figure 1). This particular biopsy was obtained via a transjugular venous access and under fluoroscopy guidance. We also pursued a coronary CTA that revealed an ectatic RCA measuring approximately 6 mm in diameter and an ectatic posterolateral branch measuring 4.5 × 5.5 mm with a fistulous communication to a dilated coronary sinus (Supplemental Videos [Supplementary-material supplementary-material-1]). The fistula was not hemodynamically significant as the patient had continued to remain asymptomatic, with normal right atrial and right ventricular dimensions and normal filling pressures on RHC. Hence, the patient did not undergo any interventions for this fistula. Despite this anomaly, the patient remained asymptomatic with improving exercise capacity on a 6-month follow-up from his heart angiogram. He was managed conservatively with good follow-up.

## 4. Discussion

The right ventricle to coronary artery fistulas are previously reported complications of EMB [9–12]. The solitary case of fistula between the coronary artery and coronary sinus has also been described [13], but in general, fistulas to cardiac veins as complication of EMB are very rare. We report a unique case of fistulous connection between the RCA and the RA via a coronary sinus. We hypothesize that in our patient, the bioptome was advanced deep into the coronary sinus and this was confused to be the right ventricular outflow tract (RVOT) on 2D fluoroscopy images. This begets a discussion about the current techniques used for EMBs, especially in light of an increased number of EMBs performed annually in the United States in recent years [6].

EMB is usually performed under fluoroscopic or 2D echocardiographic guidance with each having its advantages and pit falls [14, 15]. Owing to single plane imaging systems, most fluoroscopy techniques provide limited information about the anteroposterior movement of the bioptome tip. Furthermore, soft tissue structures are not visualized on fluoroscopy. While 2D echocardiography provides better visualization of soft tissue, it is most likely to be affected by artifacts when trying to visualize the bioptome tip. Technical advances, including 3-dimensional imaging, may allow better visualization of cardiac structures and biopsy instrumentation [16, 17]. However, the currently available data is limited and unable to definitely demonstrate superiority of one technique over the other. Imamura et al. have recommended the use of guide wire and right ventriculogram to deliver the bioptome more precisely at the site of biopsy; however, theirs is a single-center retrospective analysis with small sample size [18].

Our patient experienced a rare complication of EMB while undergoing a routine surveillance biopsy using a standard of care techniques. He was managed conservatively without any interventions for the fistula as he was asymptomatic without any evidence of right sided volume or pressure overload. However, if the fistula proved to be hemodynamically significant, we were planning on treating it with a covered stent in the coronary sinus. Although it did not prove to be clinically significant in our patient, such a fistula could ultimately lead to right sided volume overload and adverse remodeling.

Our report presents a rare and potentially hemodynamically significant complication of a routine surveillance endomyocardial biopsy. This further demonstrates the importance of careful evaluation of the biopsy site each time and using all available imaging modalities to avoid such complications.

## Supplementary Materials

Supplementary Materials Supplemental Videos 1-2: coronary angiography showing a very large RCA measuring 5 mm with a fistulous connection from posterolateral branch to the right atrium. Supplemental Video 3: TTE showing a subtle right coronary artery to right atrial shunt. Supplemental Video 4-5: coronary CTA showing an ectatic RCA measuring approximately 6 mm in diameter and an ectatic posterolateral branch measuring 4.5 × 5.5 mm with a fistulous communication to a dilated coronary sinus.
